# Dual Effects of Ketoconazole *cis-*Enantiomers on CYP3A4 in Human Hepatocytes and HepG2 Cells

**DOI:** 10.1371/journal.pone.0111286

**Published:** 2014-10-24

**Authors:** Aneta Novotná, Kristýna Krasulová, Iveta Bartoňková, Martina Korhoňová, Petr Bachleda, Pavel Anzenbacher, Zdeněk Dvořák

**Affiliations:** 1 Regional Centre of Advanced Technologies and Materials, Faculty of Science, Palacky University, Olomouc, Czech Republic; 2 Institute of Pharmacology, Faculty of Medicine and Dentistry, Palacky University, Olomouc, Czech Republic; 3 2^nd^ Department of Surgery, University Hospital Olomouc, Olomouc, Czech Republic; University of Texas Health Science Center, United States of America

## Abstract

Antifungal drug ketoconazole causes severe drug-drug interactions by influencing gene expression and catalytic activity of major drug-metabolizing enzyme cytochrome P450 CYP3A4. Ketoconazole is administered in the form of racemic mixture of two *cis*-enantiomers, i.e. (+)-ketoconazole and (−)-ketoconazole. Many enantiopure drugs were introduced to human pharmacotherapy in last two decades. In the current paper, we have examined the effects of ketoconazole *cis*-enantiomers on the expression of CYP3A4 in human hepatocytes and HepG2 cells and on catalytic activity of CYP3A4 in human liver microsomes. We show that both ketoconazole enantiomers induce CYP3A4 mRNA and protein in human hepatocytes and HepG2 cells. Gene reporter assays revealed partial agonist activity of ketoconazole enantiomers towards pregnane X receptor PXR. Catalytic activity of CYP3A4/5 towards two prototypic substrates of CYP3A enzymes, testosterone and midazolam, was determined in presence of both (+)-ketoconazole and (−)-ketoconazole in human liver microsomes. Overall, both ketoconazole *cis*-enantiomers induced CYP3A4 in human cells and inhibited CYP3A4 in human liver microsomes. While interaction of ketoconazole with PXR and induction of CYP3A4 did not display enantiospecific pattern, inhibition of CYP3A4 catalytic activity by ketoconazole differed for ketoconazole *cis*-enantiomers ((+)-ketoconazole IC_50_ 1.69 µM, K_i_ 0.92 µM for testosterone, IC_50_ 1.46 µM, K_i_ 2.52 µM for midazolam; (−)-ketoconazole IC_50_ 0.90 µM, K_i_ 0.17 µM for testosterone, IC_50_ 1.04 µM, K_i_ 1.51 µM for midazolam).

## Introduction

Ketoconazole is an imidazole antifungal drug that is used both systemically and topically, in the treatment of various fungal infections. While oral ketoconazole was discontinued in many countries, there is increasing evidence that it might be a drug of choice in the therapy of systemic infections, if the first line treatment with other antifungals fails. Various topical formulations of ketoconazole, such as creams or shampoos are massively used. Ketoconazole is also used as prophylactic agens in immune-suppressed patients (oncologic, transplant etc.). Molecular mechanism of ketoconazole action is an inhibition of fungal cytochrome P450 CYP51A, which is lanosterol-14α-demethylase that catalyzes conversion of lanosterol to ergosterol in fungi [1].

There is a myriad of potential drugs that ketoconazole can interact with, including statins, tricyclic antidepressants, antivirotics, anticonvulsives and many others. The clinical relevance of ketoconazole-drug interactions varies substantially. While certain interactions are benign and result in little or no clinical outcomes, others can produce significant toxicity or compromise efficacy if not properly managed through monitoring and dosage adjustment. Some interactions produce significant toxicity or compromise efficacy to such an extent that they cannot be managed and the particular combination of ketoconazole and interacting medicine should be avoided [2]. The mechanisms of ketoconazole-drug interactions are multiple and the most frequently, they are caused by inhibition of catalytic activity of main biotransformation enzyme CYP3A4, and also CYP2C9 [3]. Ketoconazole can disturb pharmacokinetics of drugs by interactions with transcriptional regulators of drug-metabolizing enzymes [4]. Numerous data were published regarding the effects of ketoconazole on pregnane X receptor PXR, which is a master regulator of CYP3A4 expression. We described that ketoconazole up-regulates CYP3A4 mRNA and down-regulates PXR mRNA in LS174T cells. Ketoconazole also activated CYP3A4 promoter, but it inhibited rifampicin-inducible activity of reporter gene. Binding of the ligand to PXR, and interaction of PXR with SRC1 was diminished by ketoconazole [5],[6]. Ketoconazole also blocks interactions between PXR and its transcriptional co-activator HNF4α [7]. The transcriptional activation of genes regulating biotransformation and transport by the liganded PXR was inhibited by ketoconazole. Mutations at the AF-2 surface of the human PXR ligand-binding domain indicated that ketoconazole may interact with specific residues outside the ligand-binding pocket [8]. From receptor theory point of view, this behavior indicates partial agonism of ketoconazole against PXR [9]. Structure-function as well as computational docking analysis suggested a putative binding region containing critical charge clamp residues Gln-272, and Phe-264 on the AF-2 surface of PXR. Recent study confirmed that a residue Ser-208, which is on the opposite side of the protein from the AF-2 region critical for receptor regulation, is involved in interactions between ketoconazole and PXR. The identification of new locations for antagonist binding on the surface or buried in PXR indicates novel allosteric aspects to the mechanism of receptor antagonism [10]. It was demonstrated that ketoconazole is an antagonist not only for PXR, but also for many other nuclear and steroid receptors, including glucocorticoid receptor GR [11], [12], liver X receptor LXR, constitutive androstane receptor CAR, farnesoid X receptor FXR and peroxisome proliferator-activated receptor gamma PPARγ [6]. Recently, ketoconazole was identified as an activator and ligand of aryl hydrocarbon receptor and inducer of CYP1A enzymes [12], [13].

Ketoconazole contains two chiral centers in its molecule, therefore, it forms four enantiomers. The therapeutically used ketoconazole (KET) is a racemic mixture consisting of two *cis*-enantiomers; (2R,4S)-(+)-KET and (2S,4R)-(−)-KET. Individual enantiomers of the drug may display quantitatively and/or qualitatively different pharmaco−/toxico-kinetics and pharmaco−/toxico-dynamics. The examples are numerous [14]. Logically, a concept of enantiopure drugs emerged and many enantiopure drugs were introduced to human pharmacotherapy during last two decades. Enantiospecific effects of KET on catalytic activities of CYP3A4/5 [15],[16] were reported. However, it was demonstrated that inhibition parameters of separate enantiomers for CYP3A4 are substrate-dependent and the data should be interpreted with care [17]. A phase II clinical study was conducted with compound DIO-902 (which is ketoconazole enantiomer (−)-KET), as a candidate drug for the treatment of Diabetes mellitus Type II [18]. However, due to the side effects, a study was interrupted and DIO-902 was suspended [19].

Taking in account massive use of ketoconazole, its chiral structure and numerous drug interactions, it is of value to study enantiospecific interactions between ketoconazole and drug-metabolizing pathways. We have recently described enantiospecific effects of ketoconazole *cis*-enantiomers on transcriptional activity of AhR and induction of CYP1A genes in human hepatocytes and cancer cell lines [12]. In the current paper, we have examined the effects of ketoconazole *cis*-enantiomers on the expression of CYP3A4 in human hepatocytes and HepG2 cells and on catalytic activity of CYP3A4 in human liver microsomes.

## Materials and Methods

### Compounds and reagents

Dimethylsulfoxide (DMSO), rifampicin (RIF) and hygromycin B were purchased from Sigma-Aldrich (Prague, Czech Republic). *Cis*-enantiomers of ketoconazole (2R, 4S)-(+)-KET and (2S, 4R)-(−)-KET were isolated from commercial ketoconazole by preparative HPLC at Department of Analytical Chemistry, Faculty of Science, Palacky University Olomouc. Luciferase lysis buffer was from Promega (Hercules, CA).

### Cell culture

Human Caucasian colon adenocarcinoma cells LS174T (ECACC No. 87060401) and human Caucasian hepatocellular carcinoma cells HepG2 (ECACC No. 85011430) were purchased from ECACC and were cultured in as recommended by manufacturer. Primary human hepatocytes used in this study were obtained from two sources: (i) from multiorgan donors HH52 (female; 60 years) and LH54 (male; 71 years); the use of liver cells of donors HH52 and HH54 was approved by “Ethical committee at the Faculty Hospital Olomouc”, and it was in accordance with Transplantation law #285/2002 Sb; “Ethical committee at the Faculty Hospital Olomouc” waived the authors from obtaining consent from the next of kin, regarding human hepatocytes obtained from liver donors HH52 and HH54. (ii) long-term human hepatocytes in monolayer Batch HEP220770 (female; 35 years) were purchased from Biopredic International (Biopredic International, Rennes, France). Cells were cultured in serum-free medium. Cultures were maintained at 37°C and 5% CO2 in a humidified incubator.

### mRNA determination and quantitative reverse transcriptase polymerase chain reaction

Total RNA was isolated using TRI Reagent (Molecular Research Center, Cincinnati, OH, USA). cDNA was synthesized using M-MLV Reverse Transcriptase (Finnzymes, Espoo, Finland) in the presence of random hexamers (Takara, Shiga, Japan). qRT-PCR was carried out using LightCycler FastStart DNA MasterPLUS SYBR Green I (Roche Diagnostic Corporation, Prague, Czech Republic) on a Light Cycler 480 II apparatus (Roche Diagnostic Corporation). CYP3A4 and GAPDH mRNAs were determined as described previously [20]. Measurements were performed in triplicates. Gene expression was normalized to GAPDH as a housekeeping gene.

### Protein detection and Western blotting

Total protein extracts were prepared as described elsewhere [21]. SDS-PAGE gels (10%) were run according to the general procedure followed by the protein transfer onto PVDF membrane. The membrane was saturated with 5% non-fat dried milk. Blots were probed with primary antibodies against CYP3A4 (mouse monoclonal; sc-53850, HL3) and actin (goat polyclonal; sc-1616, 1–19), both purchased from Santa Cruz Biotechnology (Santa Cruz, CA, USA). Chemiluminescent detection was performed using horseradish peroxidase-conjugated secondary antibodies (Santa Cruz Biotechnology) and Western blotting Luminol kit (Santa Cruz Biotechnology). The density of bands was measured by densitometry.

### Gene reporter assay and cytotoxicity assay

A transiently transfected LS174T human colon adenocarcinoma cells were used for assessment of PXR transcriptional activity. A chimera *p3A4-luc* reporter construct containing the basal promoter (−362/+53) with proximal PXR response element and the distal xenobiotic responsive enhancer module (−7836/−7208) of the *CYP3A4* gene 5′-flanking region inserted to pGL3-Basic reporter vector was used. The reporter plasmid was transiently transfected to LS174T cells by lipofection (FuGENE 6). Cells were incubated for 24 h with tested compounds and/or vehicle (DMSO; 0.1% v/v), in the presence or absence of RIF (10 µM; LS174T cells). After the treatments, cells were lysed and luciferase activity was measured. In parallel, cell viability was determined by conventional MTT test [MTT = 3-(4,5-dimethylthiazol-2-yl)-2,5-diphenyltetrazolium bromide]; briefly: After the treatment, culture medium was replaced with fresh one and 100 µL of MTT reagent (5 mg/mL PBS) was added. Three hours later, the medium was removed, and cells were washed with PBS and lysed for 5 min with 1 mL of DMSO containing 1% ammonia. The lysate was diluted 20 times with DMSO (+1% ammonia) and absorbance at 540 nm was measured against blank (DMSO+1% ammonia) (TECAN, Schoeller Instruments LLC). Results were normalized to the control value (i.e. 100×*A*_sample_/*A*_control_) and expressed as percentage of control.

### Catalytic activity of CYP3A4 in human liver microsomes

Chemical reagents used for microsomal incubations and HPLC analysis were purchased from commercial sources. Testosterone and 1′-hydroxymidazolam were obtained from Sigma-Aldrich CZ (Prague, Czech Republic) and 6β-hydroxytestosterone was purchased from Ultrafine (Manchester, UK). Midazolam was purchased from Abcam (Cambridge, UK).

Human liver microsomes were delivered from Xenotech (Lenexa, KS). Details of the CYP3A4/5 enzymatic activity of the mixture can be accessed from the Xenotech Web site (www.xenotechllc.com). The CYP3A4/5 activity was determined according to established protocols by using two specific substrates. Assays were based on testosterone 6β-hydroxylation and midazolam 1′-hydroxylation. Monitoring of formed metabolites from specific substrates was performed by HPLC using the Prominence system (Shimadzu, Kyoto, Japan) using reverse phase C-18 columns (LiChroCART or Chromolith-HighResolution from Merck, Darmstadt, Germany) and UV detection, according to [22]. The substrate concentrations were corresponding to the K_m_ values of measured enzymes. In the case of inhibition, K_i_ values were determined by additional measurements using extra substrate concentration (adequate to 1/2 K_m_, K_m_, 2 K_m_ and 4 K_m_). Assays were performed with eight concentrations (0.3; 1, 2, 3, 5 and 10 µM) of racemic ketoconazole and its (−)-KET and (+)-KET isomers plus ketoconazole-free controls. Incubations were performed in two independent experiments at 37°C. All reaction mixtures were buffered by 100 mM K/PO_4_ (pH 7.4) and contained an NADPH generating system consisting of isocitrate dehydrogenase, NADP^+^, isocitric acid and MgSO_4_.

Inhibition of CYP3A4/5 catalytic activities by individual ketoconazole forms was evaluated by plotting the remaining activity against the inhibitor concentration using GraphPad Prism (La Jolla, CA). The values of IC_50_ were obtained using Sigma Plot 12 scientific graphing software (SPSS, Chicago, IL). Determination of K_i_ was performed in two steps: First, the type of inhibition was assessed from Dixon plot, then, a GraphPad Prism 6 software for mixed inhibition fitted to the data points by nonlinear regression based on Henri-Michaelis-Menten equation.

### Statistics

Experiments in cell cultures were performed at least in three different cell passages. In each passage, treatments of cells were performed in triplicates. For measurement of luminescence (luciferase activity) and absorbance (MTT), triplicates from each sample were run. One-way analysis of variance followed by Dunnett’s multiple comparison post hoc test or Student’s *t* test were used for statistical analyses of data.

## Results

### Effects of ketoconazole *cis*-enantiomers on CYP3A4 mRNA and protein expression in HepG2 cells and human hepatocytes

**Figure 1:**
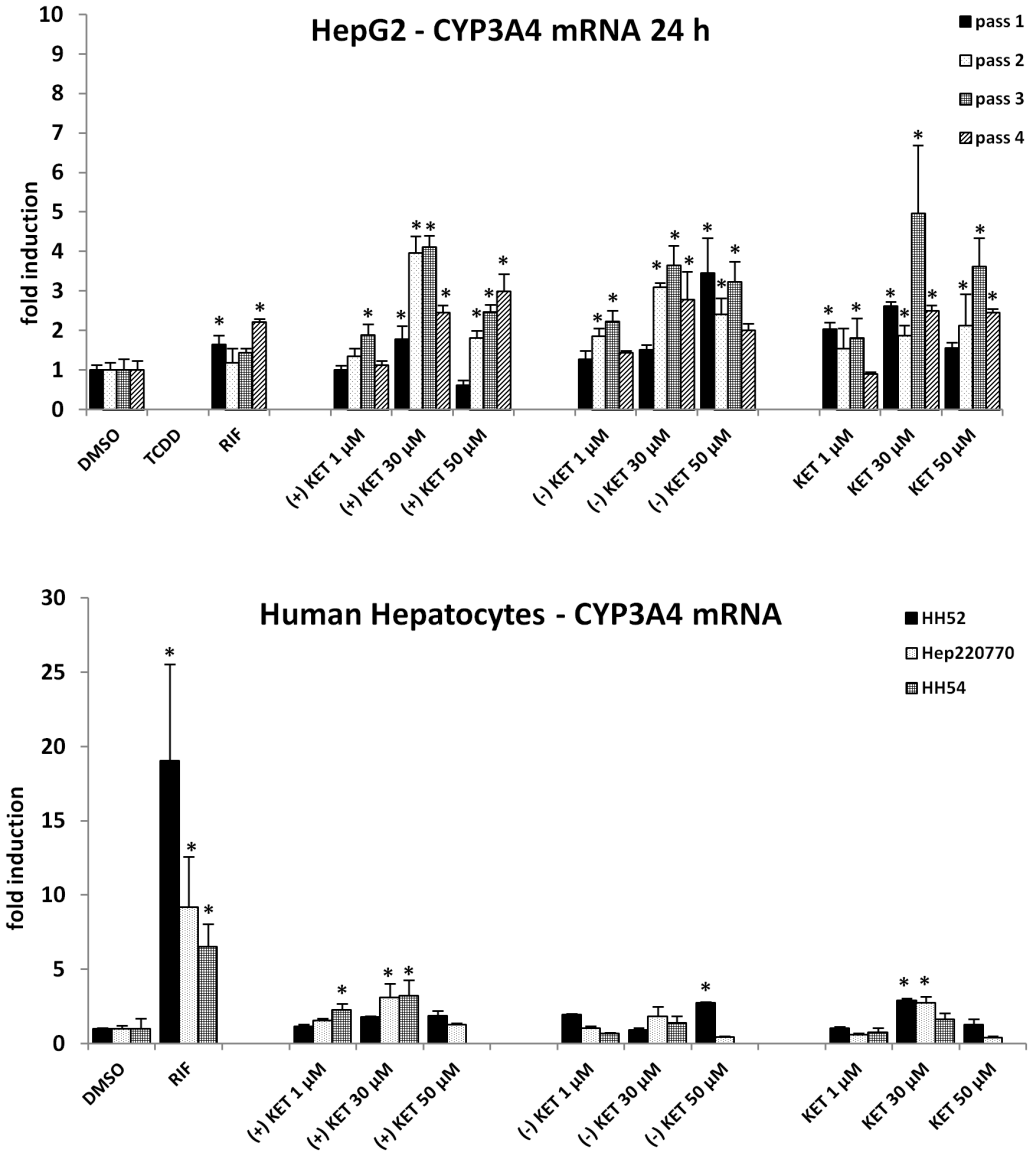
Effects of ketoconazole *cis*-enantiomers on CYP3A4 mRNA expression in HepG2 cells and human hepatocytes. (i) HepG2 cells were seeded in 6-well plates and stabilized for 16 h. Experiments were performed in four consecutive cell passages. (ii) Primary human hepatocytes from three different donors (HH52, HH54 and Hep220770) were used. Cells were incubated for 24 h with RIF (10 µM), vehicle (DMSO; 0.1% v/v) and ketoconazole ((+), (−), (rac); 1 µM, 30 µM, 50 µM). RT-PCR analyses of CYP3A4 mRNA are shown. The data are the mean ± SD from triplicate measurements and are expressed as a fold induction over vehicle-treated cells. The data were normalized to GAPDH mRNA levels. An asterisk (*) indicates that the value is significantly different from the activity of vehicle-treated cells.

**Figure 2:**
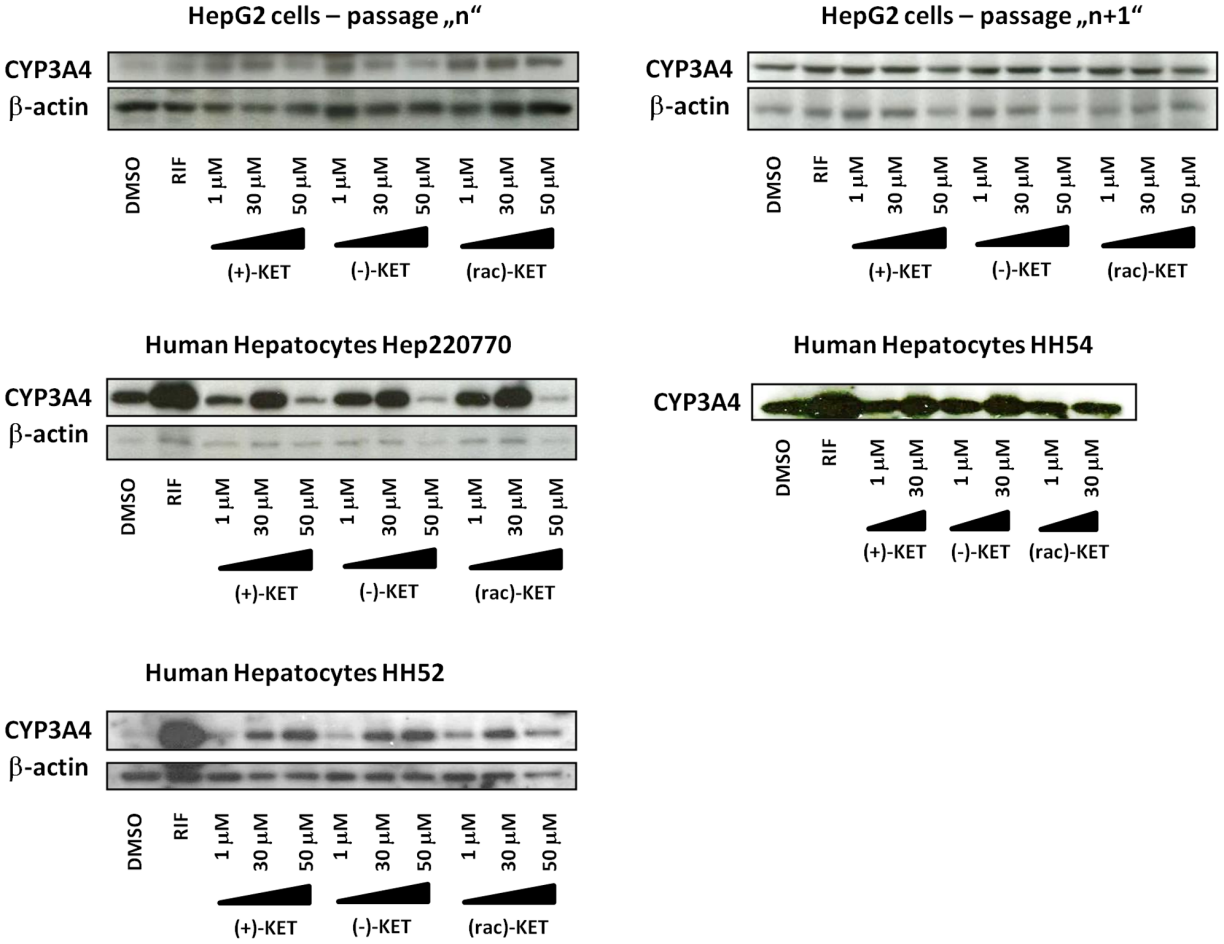
Effects of ketoconazole *cis*-enantiomers on CYP3A4 protein expression in HepG2 cells and human hepatocytes. Western blots of CYP3A4 and β-actin from three different human hepatocytes cultures (HH52, HH54 and Hep220770) and from two consecutive passages of HepG2 cells are shown. Cells were incubated for 48 h with RIF (10 µM), vehicle (DMSO; 0.1% v/v) and ketoconazole ((+), (−), (rac); 1 µM, 30 µM, 50 µM). Density of bands was quantified by densitometry.

We examined the effects of ketoconazole *cis*-enantiomers on the expression of CYP3A4 in two experimental *in vitro* systems, i.e. in human hepatoma cells HepG2 and in primary human hepatocytes. Cells were incubated for 24 h (mRNA expression) and 48 h (protein expression) with RIF (10 µM), vehicle (DMSO; 0.1% v/v) and ketoconazole ((+), (−), (rac); 1 µM, 30 µM, 50 µM). Rifampicin, a model activator of PXR and an inducer of CYP3A4 induced CYP3A4 mRNA in two of four passages of HepG2 cells by factor approx. 2-fold. All forms of ketoconazole ((+), (−), (rac)) induced CYP3A4 mRNA in HepG2 cells. The induction profiles slightly varied between four consecutive passages of HepG2 cells, and we observed either dose-dependent induction or a drop in CYP3A4 mRNA induction at 50 µM concentrations of KET, probably due to cytotoxicity issues. The effects of ketoconazole were not enantiospecific. The fold inductions of CYP3A4 mRNA by ketoconazole were comparable with those by RIF, or higher (Figure 1; upper panel). Induction of CYP3A4 mRNA by rifampicin in human hepatocytes cultures HH52, HH54 and Hep220770 was 19-fold, 7-fold and 9-fold, respectively. Induction of CYP3A4 mRNA by ketoconazole in human hepatocytes was significant (p<0.05) for following samples: culture HH52 ((−)-KET 50 µM; (rac)-KET 30 µM); culture HH54 ((+)-KET 1 µM and 30 µM); culture Hep220770 ((+)-KET 30 µM; (rac)-KET 30 µM). The induction profiles of CYP3A4 mRNA in human hepatocytes by ketoconazole were not enantiospecific and the observed differences between cultures and enantiomers are rather due to the inter-individual variability (Figure 1; lower panel). We did not find convincing induction of CYP3A4 protein in HepG2 cells incubated for 48 h with ketoconazole. All forms of ketoconazole strongly and dose-dependently (with drop of CYP3A4 protein at 50 µM of KET in some samples) induced CYP3A4 protein in three human hepatocytes cultures, but the effects were not enantiospecific (Figure 2). Overall, ketoconazole induced CYP3A4 in HepG2 cells and human hepatocytes, but the effects were not enantiospecific.

### Effects of ketoconazole *cis*-enantiomers on transcriptional activity of pregnane X receptor PXR in human LS174T gene reporter cell line

**Figure 3:**
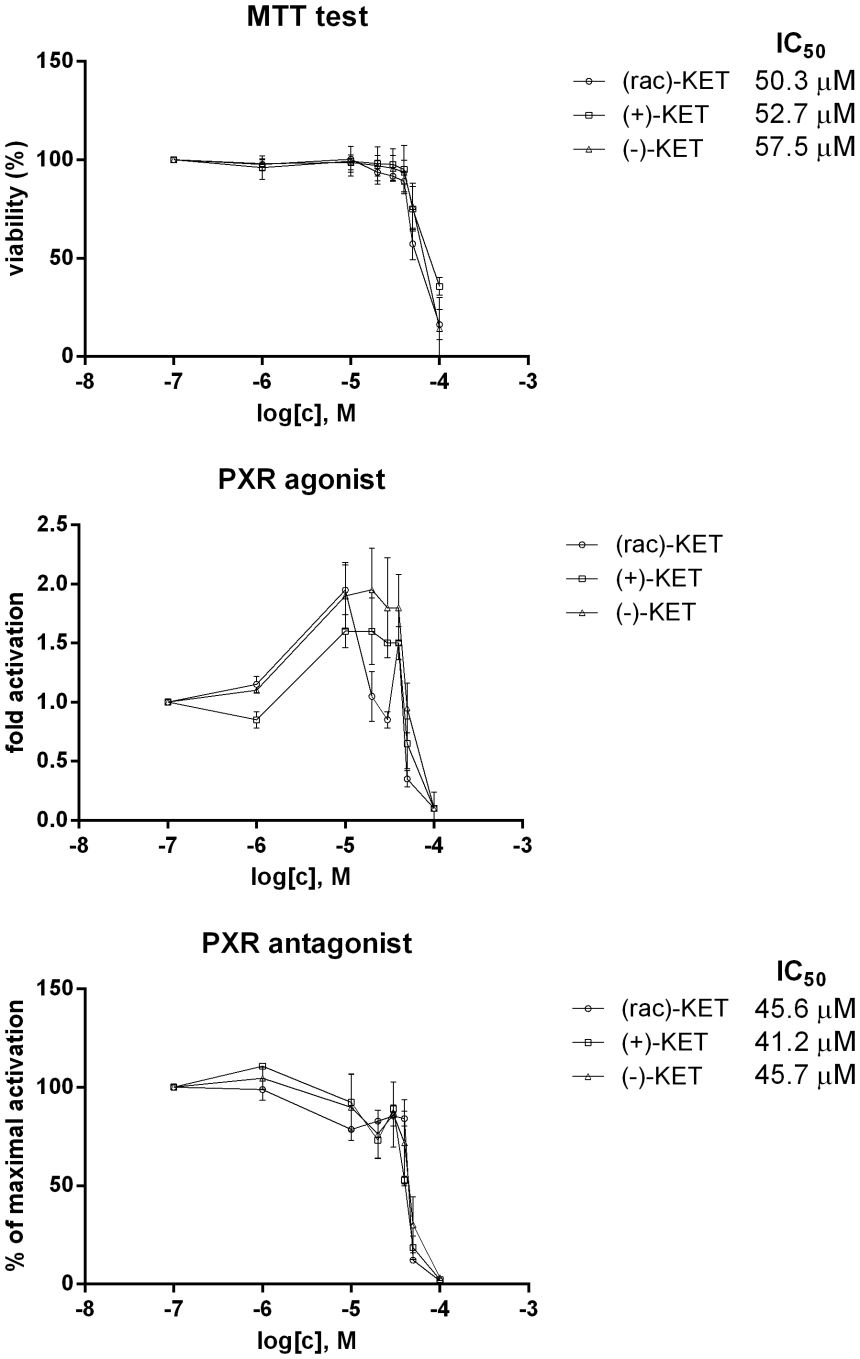
Effect of ketoconazole *cis*-enantiomers on transcriptional activity of pregnane X receptor PXR in transiently transfected LS174T cells. LS174T cells, transiently transfected with *p3A4-luc* reporter, were seeded in 24-well plates, stabilized for 16 h and then incubated for 24 h with (+)-KET, (−)-KET and rac-KET at concentrations ranging from 0.1 µM to 100 µM. The vehicle was DMSO (0.1% v/v). Model activator of PXR was rifampicin (RIF; 10 µM). Treatments were performed in triplicates. ***Upper panel:*** MTT test; The data are the mean from experiments from three consecutive passages of cells and are expressed as a percentage of viability of control cells. The values of IC_50_ were calculated and are indicated in a figure. ***Middle panel:*** *Agonist mode* - Transfected LS174T cells were incubated with KET in the absence of RIF (10 µM). The data are the mean from experiments from two consecutive passages of cells and are expressed as a fold induction of luciferase activity over control cells. ***Lower panel:*** *Antagonist mode -* Transfected LS174T cells were incubated with KET in the presence of RIF (10 µM). The data are the mean from experiments from two consecutive passages of cells and are expressed as a percentage of maximal induction attained by RIF. The values of IC_50_ were calculated and the average values are indicated in figures.

In next series of experiments, the effects of ketoconazole *cis*-enantiomers on transcriptional activity of PXR were assessed in human colon adenocarcinoma cells LS174T transiently transfected with *p3A4-luc* reporter construct. First, a cytotoxicity of tested compounds after 24 h of incubation was assessed by MTT test. We observed dose-dependent cytotoxicity of all ketoconazole forms tested, with IC_50_ values of 50.3 µM ((rac)-KET), 52.7 µM ((+)-KET) and 57.5 µM ((−)-KET). Cytotoxic effects of *cis*-enantiomers of ketoconazole were not enantiospecific (Figure 3; upper panel). In gene reporter assays, an induction of PXR-dependent luciferase activity by rifampicin varied from 20-fold to 27-fold, as compared to vehicle-treated cells. All forms of ketoconazole dose-dependently activated PXR, with maximal inductions approx. 2-fold, for 10 µM –20 µM concentrations of ketoconazole. The half-maximal effective concentrations (EC_50_) were not calculated, because of decline in luciferase activity for concentration of ketoconazole 30 µM and higher, probably due to the intrinsic cytotoxicity (Figure 3; middle panel). In antagonist mode, all forms of ketoconazole dose-dependently inhibited the activation of PXR by rifampicin. The decrease of luciferase activity was in large part caused by cytotoxic effects of ketoconazole. However, antagonistic effects of ketoconazole towards PXR were demonstrated as well, because half-maximal inhibitory concentrations IC_50_ were significantly lower as compared to those from MTT test, i.e. 45.6 µM ((rac)-KET), 41.2 µM ((+)-KET) and 45.7 µM ((−)-KET) (Figure 3; lower panel).

Collectively, ketoconazole increased basal and inhibited ligand-activated PXR transcriptional activity, and its effects were not enantiospecific.

### Effects of ketoconazole *cis*-enantiomers on catalytic activity of CYP3A in human liver microsomes

**Figure 4:**
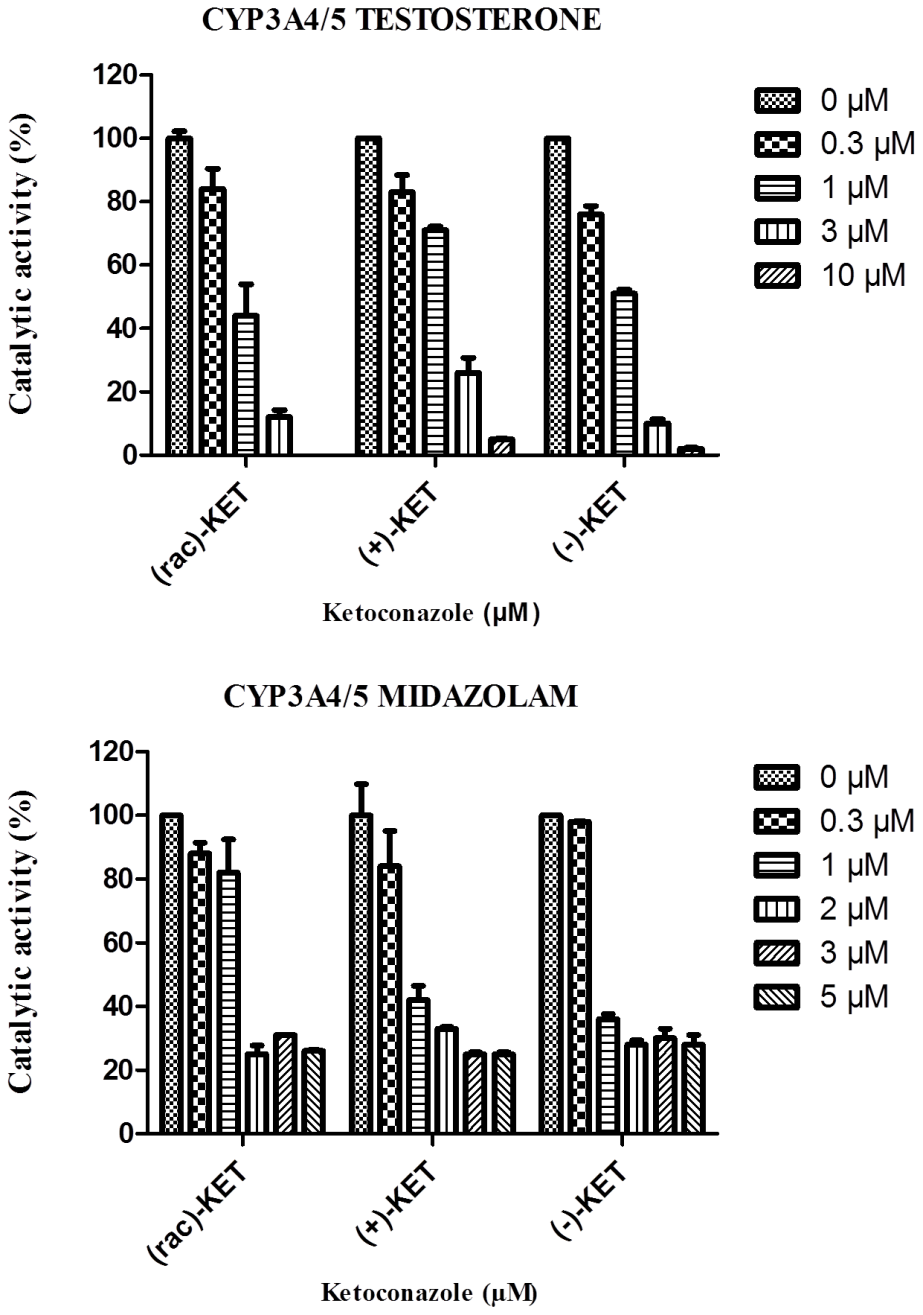
Effect of ketoconazole *cis*-enantiomers on catalytic activity of CYP3A4/5 in human liver microsomes. Inhibition of the CYP3A4/5 catalytic activity in assay with specific substrate testosterone (upper panel) and midazolam (lower panel) in human liver microsomes. Racemate, (+)-KET and (−)-KET were used in concentrations 0.3 µM, 1 µM, 2 µM, 3 µM, 5 µM and 10 µM. Inhibition of activity is determined as the mean ± SD and expressed in per cent as activity remaining relative to control (100%, without ketoconazole).

The catalytic activity of CYP3A enzymes (CYP3A4 and CYP3A5, with overlapping substrate specificity and prototypic substrates testosterone and midazolam [3] in human liver microsomes was determined to get a complete picture of the influence of ketoconazole on the properties of the CYP3A4/5 enzyme system. Both activities were reduced to values below 50%; the highest inhibition was observed with substrate testosterone (inhibition down to 3% of the initial activity at the KET concentration of 10 µM, Figure 4). To determine the K_i_ values, experiments were repeated with 4 different concentrations of substrates (corresponding to 1/2 K_m_, 1 K_m_, 2 K_m_ and 4 K_m_). The K_i_ was obtained from a nonlinear regression based on mixed type of inhibition. The IC_50_ and K_i_ data for the inhibition of CYP3A4 by racemate, (+)-KET and (−)-KET using testosterone and midazolam as substrates are shown in the Table 1. According to the IC_50_ and K_i_ values and regardless of substrate, data indicate that effect of ketoconazole is enantiospecific. The (−)-KET exhibits in all cases higher inhibitory potential than the (+)-KET. According to the result of experiment with testosterone as substrate of CYP3A, the difference between particular enantiomers is approximately 5-fold. In the case of midazolam as a specific substrate of CYP3A, the (−)-KTZ was about 1.5-fold more potent inhibitor. According to IC_50_ and K_i_, racemate usually acts similarly as (−)-KET. In this respect, the results presented here are similar to those obtained in the previous study showing the IC_50_ for inhibition of CYP3A/5-mediated testosterone and methadone metabolism by ketoconazole *cis*-enantiomers [15].

**Table 1:** Enzyme kinetics parameters for *in vitro* biotransformation two prototypic CYP3A4/5 substrates, testosterone and midazolam, with ketoconazole and two cis enantiomers, (+)-KET and (−)-KET, as inhibitors.

Ketoconazole (µM)	IC_50_ (µM)		K_i_ (µM)	
	TST	MDZ	TST	MDZ
(rac)-KET	**0.84±0.05**	**1.06±0.18**	**0.27±0.13**	**2.28±1.70**
(+)-KET	**1.69±0.27**	**1.46±0.28**	**0.92±0.47**	**2.52±1.80**
(−)-KET	**0.90±0.16**	**1.04±0.11**	**0.17±0.05**	**1.51±0.99**

TST, testosterone; MDZ, midazolam.

## Discussion

Chiral pharmacology has advanced significantly during last two decades. FDA initial guidance on chiral drugs was released in 1992, as the differential actions and toxicities of enantiomers became more evident. Racemates have virtually disappeared from development of new molecular entities. Single enantiomer or achiral drugs now dominate newly approved drugs worldwide [14]. In addition, racemic drugs previously granted patents are expiring, so they become candidates for a “chiral switch”, i.e. development of single enantiomer or paired enantiomers in case of diastereomers [23]. This permits additional years of market exclusivity, and pharmaceutical companies are sometimes suspected from “evergreening” the drugs. Ideally, therapeutic activity would reside in one enantiomer (eutomer) and adverse effects in the other (dystomer). However, there is a range of possibilities, and the combined actions of the individual enantiomers may actually make the racemate or enantiomer combinations even desirable [14].

In the current paper, we have studied the effects of ketoconazole *cis*-enantiomers on the expression and catalytic activity of CYP3A4 in human *in vitro* systems. Ketoconazole exists in the form of four enantiomers. The therapeutically used ketoconazole is a racemic mixture consisting of two *cis*-enantiomers; (2R,4S)-(+)-KET and (2S,4R)-(−)-KET. Since numerous ketoconazole-drug interactions were reported, it is certainly of value to investigate, whether one of the ketoconazole enantiomers exerts less interaction potential against CYP3A4 as compared to other enantiomer. We have recently examined antifungal activities of ketoconazole *cis*-enantiomers against 7 strains of *Candida spp.* and we demonstrated that (+)-KET is two times more potent than (−)-KET for strains *C. albicans* and *C. tropicalis,* while (−)-KET is seven times more potent than (+)-KET for other five tested strains [12], which was in line with observations of other authors [24]. These data imply enantiospecific pattern of clinical (desirable) activities of ketoconazole. We have also observed enantiospecific effect of ketoconazole on AhR-CYP1A signaling pathway, the activation of which may be considered as undesired effect of ketoconazole [12]. On the other hand, antagonistic effects of ketoconazole against glucocorticoid receptor GR were not enantiospecific [12]. In the present paper we show that ketoconazole partial agonist activity against pregnane X receptor PXR (Figure 3), as well as ketoconazole-mediated induction of CYP3A4 (Figure 1, Figure 2) are not enantiospecific, i.e. both *cis*-enantiomers ((+), (−)) were equipotent.

As the aim of this paper was to examine the possible enantiospecific differences in the interactions of the KET *cis*-enantiomers with the CYP3A enzyme system, we studied also the possible changes in the inhibition of CYP3A enzyme in human liver microsomes by these compounds. Ketoconazole is generally considered as CYP3A4/5 -specific inhibitor [25]. According to our results, as well as by data from the literature [15], [16], both the (+)-KET and (−)-KET exhibit distinct, moderate differences in the values of the IC_50_ parameters characterizing the testosterone, alprazolam, quinidine and midazolam metabolism with the (−)-KET being more potent. Here, for prediction of the drug-drug interaction mediated by (−)- KET and (+)-KET by CYP3A in more detail, the K_i_ values of *cis*-enantiomers were estimated for the first time. On the other hand, there are several studies discussing inhibition of CYP3A by racemic ketoconazole; the extent of inhibition is usually expressed both as the IC_50_ and K_i_. It is however known that the inhibitory potency of ketoconazole is highly variable (K_i_ values may range, for different CYP3A substrates, from 0.001 to 25 µM) [3]. In this study, we have found the K_i_ for racemic KET 0.27 µM for testosterone 6β-hydroxylation and 2.28 µM for midazolam 1′-hydroxylation, which fits into this range. In general, the differences may be caused by various factors. The most important factor is apparently an incorrect assignment of inhibition mechanism. According to our data analysis, the mixed model of inhibition mechanism was used which was found also by another study [25]. For the (−)-KET, 5-fold stronger inhibitory potency in testosterone metabolism and 1.5-fold in case of midazolam was found. Racemate acted similarly as the (−)-KET, being, in other words, more potent inhibitor of CYP3A4/5 than the (+)-KET. Hence, for the ability to inhibit the CYP3A enzymes, the (−)-KET enantiomer is more responsible. Interestingly, as it has been shown in our previous paper (discussed above), antifungal activity of *cis*-enantiomers on several strains was determined with a result describing enantiospecificity of the biological effect of this drug [12]. The (−)-KET seems to be more potent inhibitor of CYP51 in most fungi. In conclusion, the enzyme activity data presented in this paper, support enantiospecific clinical pattern of ketoconazole effect.

In conclusion, we show that ketoconazole inhibits enantiospecifically CYP3A4/5 catalytic activity, while its effects of CYP3A4 expression and PXR activity are not enantiospecific. Regarding PXR-CYP3A4 signaling and metabolic cascade, the potential enantiopure preparations of ketoconazole in human pharmacotherapy are not of interest.

## References

[1] Heeres J, Meerpoel L, Lewi P (2010) Conazoles. Molecules 15: 4129–4188. 20657432 10.3390/molecules15064129 PMC6264770

[2] Gubbins PO, Heldenbrand S (2010) Clinically relevant drug interactions of current antifungal agents. Mycoses 53: 95–113. 20002883 10.1111/j.1439-0507.2009.01820.x

[3] Greenblatt DJ, Venkatakrishnan K, Harmatz JS, Parent SJ, von Moltke LL (2010) Sources of variability in ketoconazole inhibition of human cytochrome P450 3A in vitro. Xenobiotica 40: 713–720. 20712450 10.3109/00498254.2010.506224

[4] Dvorak Z (2011) Drug-drug interactions by azole antifungals: Beyond a dogma of CYP3A4 enzyme activity inhibition. Toxicol Lett 202: 129–132. 21333771 10.1016/j.toxlet.2011.01.027

[5] Svecova L, Vrzal R, Burysek L, Anzenbacherova E, Cerveny L, et al (2008) Azole antimycotics differentially affect rifampicin-induced pregnane X receptor-mediated CYP3A4 gene expression. Drug Metab Dispos 36: 339–348. 17998298 10.1124/dmd.107.018341

[6] Huang H, Wang H, Sinz M, Zoeckler M, Staudinger J, et al (2007) Inhibition of drug metabolism by blocking the activation of nuclear receptors by ketoconazole. Oncogene 26: 258–268. 16819505 10.1038/sj.onc.1209788

[7] Lim YP, Kuo SC, Lai ML, Huang JD (2009) Inhibition of CYP3A4 expression by ketoconazole is mediated by the disruption of pregnane X receptor, steroid receptor coactivator-1, and hepatocyte nuclear factor 4alpha interaction. Pharmacogenet Genomics 19: 11–24. 19077665 10.1097/FPC.0b013e32831665ea

[8] Wang H, Huang H, Li H, Teotico DG, Sinz M, et al (2007) Activated pregnenolone X-receptor is a target for ketoconazole and its analogs. Clin Cancer Res 13: 2488–2495. 17438109 10.1158/1078-0432.CCR-06-1592

[9] Venkatesh M, Wang H, Cayer J, Leroux M, Salvail D, et al (2011) In vivo and in vitro characterization of a first-in-class novel azole analog that targets pregnane X receptor activation. Mol Pharmacol 80: 124–135. 21464197 10.1124/mol.111.071787 PMC3127530

[10] Li H, Redinbo MR, Venkatesh M, Ekins S, Chaudhry A, et al (2013) Novel yeast-based strategy unveils antagonist binding regions on the nuclear xenobiotic receptor PXR. J Biol Chem 288: 13655–13668. 23525103 10.1074/jbc.M113.455485 PMC3650402

[11] Duret C, Daujat-Chavanieu M, Pascussi JM, Pichard-Garcia L, Balaguer P, et al (2006) Ketoconazole and miconazole are antagonists of the human glucocorticoid receptor: consequences on the expression and function of the constitutive androstane receptor and the pregnane X receptor. Mol Pharmacol 70: 329–339. 16608920 10.1124/mol.105.022046

[12] Novotna A, Korhonova M, Bartonkova I, Soshilov AA, Denison MS, et al (2014) Enantiospecific Effects of Ketoconazole on Aryl Hydrocarbon Receptor. PLoS One 9: e101832. 25000292 10.1371/journal.pone.0101832 PMC4084896

[13] Korashy HM, Shayeganpour A, Brocks DR, El-Kadi AO (2007) Induction of cytochrome P450 1A1 by ketoconazole and itraconazole but not fluconazole in murine and human hepatoma cell lines. Toxicol Sci 97: 32–43. 17283379 10.1093/toxsci/kfm012

[14] Smith SW (2009) Chiral toxicology: it's the same thing…only different. Toxicol Sci 110: 4–30. 19414517 10.1093/toxsci/kfp097

[15] Dilmaghanian S, Gerber JG, Filler SG, Sanchez A, Gal J (2004) Enantioselectivity of inhibition of cytochrome P450 3A4 (CYP3A4) by ketoconazole: Testosterone and methadone as substrates. Chirality 16: 79–85. 14712470 10.1002/chir.10294

[16] Allqvist A, Miura J, Bertilsson L, Mirghani RA (2007) Inhibition of CYP3A4 and CYP3A5 catalyzed metabolism of alprazolam and quinine by ketoconazole as racemate and four different enantiomers. Eur J Clin Pharmacol 63: 173–179. 17200836 10.1007/s00228-006-0230-z

[17] Stresser DM, Blanchard AP, Turner SD, Erve JC, Dandeneau AA, et al (2000) Substrate-dependent modulation of CYP3A4 catalytic activity: analysis of 27 test compounds with four fluorometric substrates. Drug Metab Dispos 28: 1440–1448. 11095581

[18] Schwartz SL, Rendell M, Ahmann AJ, Thomas A, Arauz-Pacheco CJ, et al (2008) Safety profile and metabolic effects of 14 days of treatment with DIO-902: results of a phase IIa multicenter, randomized, double-blind, placebo-controlled, parallel-group trial in patients with type 2 diabetes mellitus. Clin Ther 30: 1081–1088. 18640464 10.1016/j.clinthera.2008.05.021

[19] Arakaki R, Welles B (2010) Ketoconazole enantiomer for the treatment of diabetes mellitus. Expert Opin Investig Drugs 19: 185–194. 10.1517/13543780903381411 20047506

[20] Vrzal R, Knoppova B, Bachleda P, Dvorak Z (2013) Effects of oral anorexiant sibutramine on the expression of cytochromes P450s in human hepatocytes and cancer cell lines. J Biochem Mol Toxicol 27: 515–521. 24038852 10.1002/jbt.21516

[21] Novotna A, Srovnalova A, Svecarova M, Korhonova M, Bartonkova I, et al (2014) Differential effects of omeprazole and lansoprazole enantiomers on aryl hydrocarbon receptor in human hepatocytes and cell lines. PLoS One 9: e98711. 24887303 10.1371/journal.pone.0098711 PMC4041848

[22] Phillips IR Shephard EA, editors (2006) Cytochrome P450 Protocols. 2nd ed. Totowa: Humana Press.

[23] Caner H, Groner E, Levy L, Agranat I (2004) Trends in the development of chiral drugs. Drug Discov Today 9: 105–110. 15038394 10.1016/s1359-6446(03)02904-0

[24] Rotstein DM, Kertesz DJ, Walker KA, Swinney DC (1992) Stereoisomers of ketoconazole: preparation and biological activity. J Med Chem 35: 2818–2825. 1495014 10.1021/jm00093a015

[25] Greenblatt DJ, Zhao Y, Venkatakrishnan K, Duan SX, Harmatz JS, et al (2011) Mechanism of cytochrome P450-3A inhibition by ketoconazole. J Pharm Pharmacol 63: 214–221. 21235585 10.1111/j.2042-7158.2010.01202.x

